# Decreased, but still sufficient, iodine intake of children and adults in the Netherlands

**DOI:** 10.1017/S0007114517000733

**Published:** 2017-04-14

**Authors:** Janneke Verkaik-Kloosterman, Elly J. M. Buurma-Rethans, Arnold L. M. Dekkers, Caroline T. M. van Rossum

**Affiliations:** National Institute for Public Health and the Environment (RIVM), PO Box 1, 3270 BA Bilthoven, The Netherlands

**Keywords:** Iodine, Dietary sources, Adults, Children, Habitual intake

## Abstract

Sufficient I intake is important for the synthesis of thyroid hormones, which play an important role in normal growth and development. Our aim was to estimate habitual I intake for the Dutch population and the risk of inadequate or excessive intakes. Further, we aimed to provide an insight into the dietary sources of I and the association with socio-demographic factors. Data from the Dutch National Food Consumption Survey 2007–2010 (*n* 3819; 7–69 years), and from the Dutch food and supplement composition tables were used to estimate habitual I intake with a calculation model. Contribution of food groups to I intake were computed and multiple linear regression was used to examine associations of intakes with socio-demographic factors. A total of ≤2 % of the population had an intake below the estimated average requirement or above the upper level. The main sources of I were bread containing iodised salt (39 %), dairy products (14 %) and non-alcoholic drinks (6 %). I intake (natural sources only, excluding iodised salt and supplements) was positively associated with (parental) education, which could at least partly be attributed to a higher consumption of dairy products. Among children, the consumption of bread, often containing iodised bakery salt, was positively associated with parental education. The I intake of the Dutch population (7–69 years) seems adequate, although it has decreased since the period before 2008. With the current effort to reduce salt intake and changing dietary patterns (i.e. less bread, more organic foods) it is important to keep a close track on the I status, important sources and potential risk groups.

I is an essential element for efficient functioning of the thyroid gland and the synthesis of thyroid hormones. These hormones play an important role in the early growth and development of several organs, especially the brain; further, they regulate metabolic processes. Inadequate I intake results in I deficiency disorders, with goitre as a well-known clinical sign. In addition, I deficiency during early childhood, including pregnancy, results in impaired brain development and consequently, reduced mental functioning^(^ ^1^ ^)^. Besides harmful effects of too low I intakes, excessive intakes cause biochemical changes such as elevated thyroxin and decreased thyroid stimulating hormone concentrations. It is, however, uncertain if clinical health effects would appear because of chronic exposure to these biochemical changes^(^ ^2^ ^)^.

In many countries, including the Netherlands, the natural I levels of foods are not adequate. Therefore, worldwide, I fortification and supplementation programmes are implemented^(^ ^1^ ^)^. Nevertheless, in many countries including Europe, (mild/moderate) I deficiency remains a public health problem^(^ ^3^ ^)^. The Netherlands has a long history of salt iodisation programmes starting in 1928 (Box 1), and is considered to be one of the countries with adequate I intakes^(^ ^1^ ^,^ ^4^ ^,^ ^13^ ^)^.

Box 1 Historic overview of iodisation programmes in the Netherlands^(^ ^4^ ^–^ ^12^ ^)^

**Table:** 

Years	Event
1928	Introduction iodised kitchen salt, mainly used on medical advice
1932	Regional iodisation of drinking water (50 µg KI/l); because of introduction of tap water, an increase in goitre in some regions
1942	Introduction of iodised bread salt (31 mg I/kg salt) and stop of regional iodisation of drinking water
1960	Start of mandatory iodisation of bread salt (39 mg KI/kg salt) in regions with low water I concentration (<40 µg/l)
1968	Mandatory iodisation of bread salt (46 mg KI/kg salt) across the whole country: ‘Broodbesluit’
1974	Iodised kitchen salt no longer available
1982	I level in bread salt increased to 60 mg KI/kg and reintroduction of iodised kitchen salt (23–29 mg KI/kg): ‘Zoutbesluit’
1984	Court decision: mandatory iodisation of bread salt replaced by voluntary addition regulated via a covenant between the Minister of Health and the (industrial) bakeries
1985	I level in bread salt revised to 55–65 mg KI/kg
1998	New legislation: addition of I to foods is prohibited except for bread salt (55–65 mg KI/kg salt) and kitchen salt (45–55 mg KI/kg)
1999	Revision I legislation: kitchen salt 30–40 mg KI/kg, bread salt 70–85 mg KI/kg, nitrate grid salt in meat products 30–40 mg KI/kg
2004	Arrest EU court: bread with lower I levels permitted, otherwise a trade barrier
2008	Revision I legislation: bakery salt (for all bakery products) maximum 65 mg KI/kg, salt for other products (including kitchen salt) maximum 25 mg KI/kg

The national I policy changed several times in the Netherlands, to create or maintain the population’s adequate I intake, to prevent excessive intake and to meet European legislation (Box 1). The most recent change is from 2008^(^ ^10^ ^)^. Before 2008, the addition of iodised salt was limited to specific food groups (bread, household salt, bread-replacing products, meat products) at specified levels (bread salt 70–85 mg I/kg salt; household salt 30–40 mg I/kg salt; meat products 20–30 mg I/kg salt). For all these food groups, the addition of iodised salt was on a voluntary basis; however, there is a covenant between the Ministry of Health and the bakery sector to use iodised bread salt for bread (because of a court decision (Box 1) organic bread is excluded from this covenant, although iodised bread salt may voluntary be added to organic bread). Since 2008, iodised bakery salt (a maximum of 65 mg I/kg salt) may be added to bread and all bakery products, still on a voluntary basis. For all other food, iodised salt with a maximum of 25 mg I/kg salt may be used. With the change of the I policy, it was expected that 50 % of the foods other than bread would contain iodised salt^(^ ^14^ ^)^. Because of the covenant between the Ministry of Health and the bakery sector, almost all bread contains iodised bakery salt^(^ ^5^ ^)^.

The I status was measured in 2006 and 2010 in a cohort in the Netherlands. Estimates of the I intake from non-replicated 24-h urine samples showed a decrease in I intake; in 2010 the median intake was 179 *v*. 262 µg/d in 2006^(^ ^13^ ^)^. In spite of the change in the Dutch I policy, a decrease in I intake was not anticipated. Although measurement of repeated 24-h urinary I excretion in combination with statistical correction for within-person variation is the best way to get an insight into the habitual I intake and status^(^ ^15^ ^–^ ^17^ ^)^, such data do not provide information on major I sources in the population’s diet and does not give the opportunity to estimate the effects of changing I and/or salt policies on the intake. Earlier, Verkaik-Kloosterman *et al.* ^(^ ^18^ ^,^ ^19^ ^)^ developed a model to estimate the habitual I intake from food consumption survey data collected with two repeated 24-h recalls per subject. The aims of the present study were to assess the adequacy and safety of I intake in the Dutch population, to get an insight into the main sources of I in the Dutch diet and understand the socio-demographic factors affecting the I intake. An insight into socio-demographic factors associated with I intake can be used to improve interventions or I policy. With a slightly adapted version of this model, the habitual I intake was estimated for the Dutch population using data from the most recent Dutch National Food Consumption Survey (DNFCS) 2007–2010 and data from the Dutch food and supplement composition tables. The adequacy and safety of I intake in the Netherlands was evaluated by estimation of the proportion of the population with intakes below the estimated average requirement (EAR) set by the Institute of Medicine^(^ ^20^ ^)^ or above the Tolerable upper intake level (UL) set by the Scientific Committee on Food (SCF)^(^ ^2^ ^)^.

## Methods

### Survey population

Data from the DNFCS 2007–2010 were used, in which the dietary intake was estimated from two non-consecutive 24-h recalls. Details of this representative cross-sectional study are described elsewhere^(^ ^21^ ^)^; in brief, subjects were drawn from representative consumer panels of Market Research GfK Panel Services. Data were collected among Dutch children and adults aged 7–69 years (study sample *n* 3819; net response rate 69 %); pregnant and lactating women as well as institutionalised people were excluded. The sampling was guided by socio-demographic characteristics and stratified by age and sex. The distribution of levels of education, region and urbanisation in the study population were close to the general Dutch population. Days of the week and seasons were almost equally spread, with a small overrepresentation of winter, and Tuesdays and Thursdays at the expense of Saturdays. For the small deviations of the survey population from the Dutch population, a weighing factor was created based on socio-demographic characteristics, season and day of the week. Using this weighing factor, the results can be considered representative for the Dutch population over a calendar year. This weighting factor was applied in all statistical analyses. Unless otherwise stated, all analyses were performed with SAS 9.2 (SAS Institute Inc.). The study was conducted according to the guidelines of the Helsinki Declaration.

### Dietary assessment

Detailed information on food and dietary supplement consumption was collected by trained dietitians using the computer-assisted 24-h recall method – GloboDiet (formerly EPIC-Soft^®^ of the International Agency for Research on Cancer). All subjects were interviewed on 2 non-consecutive days (2–6 weeks apart). Children aged 7–15 years were interviewed face-to-face during (appointed) home visits in presence of the parent/caretaker. Persons aged ≥16 years were interviewed by telephone at unannounced dates and times. Interviews were not planned on national and/or religious holidays, or when the participant was on holiday. In addition to the 24-h recalls, lifestyle characteristics were collected with a general questionnaire, including general characteristics of the dietary pattern and the consumption of specific foods and supplements. Height and weight were self-reported by the subjects.

The I concentration naturally present in foods were taken from the Dutch Food Composition Database (NEVO-online 2011/3.0)^(^ ^22^ ^)^. This database contains information of more than 2000 foods, preferably from chemical analyses, supplemented with information collected from foreign food composition tables, scientific literature and food labels. The source of each composition value is known and can be found in NEVO-online. For the composition of supplements, the Dutch Supplement Database of 1 January 2008 (NES) was used^(^ ^23^ ^)^.

### Estimation total habitual iodine intake distribution

The total I intake can be divided into four main sources, namely:

(a) I naturally present in food (excluding iodised salt and supplements);
(b) iodised salt used by food manufacturers;
(c) iodised salt used during meal preparation and consumption; and
(d) dietary supplements.

**Fig. 1:**
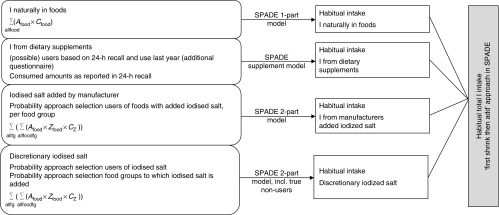
Schematic overview of the mathematical model to estimate the habitual total iodine intake distribution. Food, consumed food; *A*_food_, consumed amount of a food; *C*_food_, iodine concentration in a food; fg, food group; *C*_z_, iodine concentration in salt; *Z*_food_, salt (added sodium) concentration in a food; SPADE, Statistical Program to Assess Dietary Exposure

We used an adapted version of the model described by Verkaik-Kloosterman *et al.* ^(^ ^18^ ^,^ ^19^ ^)^. In short, the habitual I intake distribution was estimated separately, using an age-dependent model, for each of the four main sources; thereafter, these four distributions were combined using Monte Carlo simulation to get the total habitual I intake distribution (Fig. 1). Habitual intake was defined as the long-time average intake of an individual and was estimated using the Statistical Program to Assess Dietary Exposure (SPADE version 3.1 of 27 November 2015)^(^ ^26^ ^)^. On the basis of 2 independent survey d per subject, the data can be corrected for the within-person variation to get the population’s habitual intake distribution.

#### (a) Iodine naturally present in food (excluding iodised salt and supplements)

The intake of I naturally present in foods (excluding iodised salt and supplements) (a) was estimated by combining the food consumption data with the food composition data, resulting in the I intake per food item (NEVO-code) per subject per survey day. To estimate the contribution of several food groups to the intake of I naturally present in foods, the mean intake over 2 survey d was estimated for each subject. Per food group, the distribution of the proportion of intake of I naturally present in foods was calculated. Bread and other foods known to be produced with iodised salt were not included in this category, but taken into account in the category (b) iodised salt used by food manufacturers. The habitual intake distribution was estimated.

#### (b) and (c) Iodised salt added during processing or during meal preparation and consumption

Detailed information on the discretionary use of iodised salt (c) and the application of iodised salt by food manufacturers (b) is lacking; therefore, the total I intake was estimated using a probabilistic calculation model, in order to quantify the uncertainty. From the survey population (*n* 100; Monte Carlo sampling), samples were repeatedly drawn (stratified by age and sex) to assign a subject as discretionary user of salt. From each of these samples, an additional sample was drawn to assign discretionary users of salt to the use of iodised salt. A similar procedure was applied for the estimation of I intake from iodised salt added by food manufacturers; however, the sampling (*n* 100; Monte Carlo sampling) was performed among consumers of specific food groups (Table 1), instead of the whole population. Samples were drawn separately for different foods or food groups, and selected subjects were assigned to consume foods from that specific food group containing iodised salt. The proportion of subjects drawn was equal to the proportions presented in Table 1. This resulted in 100 distributions of I intake from discretionarily added iodised salt as well as iodised salt added by the food manufacturer. The differences between these 100 distributions were taken to represent the uncertainty in estimation of the I intake from these main sources. The habitual intake distribution was estimated with SPADE for each of these 100 samplings, resulting in 100 habitual intake distributions for the discretionarily added I intake as well as the intake of I from iodised salt added by manufacturers. For I intake from iodised salt added by manufacturers a SPADE 2-part model^(^ ^24^ ^)^ was applied, as a part of the consumers of the selected foods were selected to have consumed products without added iodised salt, resulting in a zero intake from this source. For discretionarily added I, the habitual intake was estimated with a SPADE 2-part model taking into account true non-consumers^(^ ^24^ ^)^ of this source, that is, subjects never adding salt to their food during meal preparation or consumption.

**Table 1:** Overview of assumptions in the calculation model to estimate habitual total iodine intake in the Dutch population based on data from the Dutch National Food Consumption Survey (DNFCS) 2007–2010

	Assumption	
Descriptions	Proportion with no salt use (%)	If salt; proportion with no iodised salt (%)
Reported discretionary use of (iodised) salt*		
Age group (years)		
7–8		
Males	16	9
Females	15	15
9–13		
Males	7	9
Females	16	15
14–18		
Males	5	8
Females	8	10
19–30		
Males	13	11
Females	14	11
31–50		
Males	9	21
Females	16	19
51–69		
Males	17	8
Females	17	6
Discretionary use of salt per food group†	Proportion (%)	g salt/100 g
Food group		
Potatoes	85	0·4
Mashed potatoes	85	0·6
Rice and pasta	85	0·4
Vegetables	75	0·6
Meat	95	1·8
Fish	95	1·8
Meat replacers	95	1·8
Eggs	75	1·8
Homemade sauces	80	0·8
Pancakes	80	0·2
Use of iodised salt by manufacturers‡	Proportion (%)	Low/high iodised salt§
Product group		
Bread	95	High
Pizza	40	Low
Pastry and cookies	1	High
Dutch rusk	0·5	Low
Crackers	0·5	Low
(Sliced) cold meat	0·5	Low

* Calculated from data of 3rd year of data collection DNFCS 2007–2010.

† Foods containing salt added by manufacturers were excluded.

‡ For branded foods with known use of iodised salt a deterministic approach was applied. Baking mix, chips for children, dressing and sausage rolls are available with iodised salt; however, the number of users in DNFCS 2007–2010 was <100; using a market share of 0·5 %, this would result in <1 person; these products were considered as never containing iodised salt.

§ High iodised salt: bakery salt containing on average 58 mg I/kg salt; low iodised salt: on average 20 mg I/kg salt.

#### (d) Dietary supplements

In DNFCS, potential users of I-containing dietary supplements were defined as follows: subjects who reported intake of I from dietary supplements in at least one of the 24-h recalls and/or subjects who reported use of supplement categories that may contain I in the frequency questionnaire. Consequently, for some subjects no amount information was available (user according to frequency questionnaire, but no intake in 24-h recalls). For subjects with a known amount of I intake from dietary supplements, these amounts were set as the habitual intake amount, as described by Dekkers *et al*. For the remaining subjects having no I intake from dietary supplements on the recall days, the habitual intake amount was predicted with a regression tree model^(^ ^24^ ^)^. The SPADE supplement model was used to estimate the habitual I intake from dietary supplements^(^ ^24^ ^)^.

### Assumptions for iodine intake from iodised salt

As stated above, detailed information on the use of iodised salt is lacking; therefore, additional information was gathered as input in the probabilistic approach. In the 3rd year of data collection of DNFCS 2007–2010 the additional questionnaire was expanded with questions on the use of (iodised) salt during meal preparation and consumption. From this information, the proportion of subjects that answered to never use discretionarily (iodised) salt was calculated (stratified by age and sex) (Table 1). These proportions were used for the sampling of subjects in the 1st and 2nd years of data collection. Per age and sex category, a sample as large as the proportion was drawn for 100 iterations. The selected subjects were assigned to not discretionarily adding (iodised) salt. The remaining subjects were assigned to discretionarily adding (iodised) salt. For the 3rd year of data collection, a deterministic approach was applied using the subject-specific information directly.

The discretionary use of (iodised) salt varies among food groups^(^ ^25^ ^)^ (D van der Zee, unpublished results, Dutch Bakery Center (NBC), 16 September 2011). In the current calculation model, it was therefore not assumed that subjects discretionarily adding (iodised) salt would do this always for all food groups they consumed, in contrast with the earlier calculation model^(^ ^18^ ^,^ ^19^ ^)^. Unfortunately, there are no data available on the discretionary use of (iodised) salt for different food groups that distinguish between foods produced with and those produced without salt. In the calculation model, foods produced with salt were not selected for discretionary addition of salt either. On the basis of the proportions of subjects using (iodised) salt in the 3rd year of data collection in DNFCS 2007–2010 and the very limited information on differences in salt use between food groups^(^ ^25^ ^)^ (D van der Zee, unpublished results, NBC, 16 September 2011), the authors made an assumption for the proportion of subjects discretionarily adding salt to the food groups potatoes, rice and pasta, vegetables, meat and meat replacers, eggs, fish, homemade sauces and pancakes (Table 1). The amount of salt discretionarily added was taken from Verkaik-Kloosterman *et al*.^(^ ^18^ ^,^ ^19^ ^)^. In these studies, the amount of salt added per 100 g food was estimated on the basis of cookbook recipes and guidelines of the Dutch Food Consumption Table.

There is no up-to-date information on the use of iodised salt by Dutch food manufacturers. For our study the Federation of Dutch grocery and food industry (FNLI) and the Dutch Bakery Association (NVB) have sent a questionnaire to all their members to find out how often iodised salt was used. Most of the fresh bread was prepared with iodised bakery salt, as well as about half of the partially baked bread segment (WM van Andel (NVB) and C Grit (FNLI), unpublished results, 8 December 2011). On the basis of market shares and the fact that part of the organic bread is prepared with non-iodised sea salt, it was assumed that 95 % of the consumed bread contained iodised salt. In addition, several specific brands of breakfast crackers and gingerbreads contained iodised salt. As these foods were present in NEVO with a specific NEVO-code, the I content was added to this code. Also two big pizza brands, having a market share of about 40 %, use iodised salt. Salt (Na) in pizza originates from many different sources, for example, the dough, the various toppings, the cheese. Not all the Na in pizza is from added salt; in addition, not all different parts of the pizza may be processed with iodised salt; therefore, it was assumed by the authors that, on average, 50 % of the salt in pizza is from added iodised salt. Besides this questionnaire, the INNOVA database (www.innovadatabase.com) was searched for foods available in the Netherlands containing iodised salt as an ingredient. This showed that foods imported mainly from Germany contain iodised salt. In addition, it showed that foods produced with iodised salt are available in many different food groups, however the proportion within each food group is in general limited. On the basis of this information, the market shares were assumed to be 0·5–1 %. In DNFCS 2007–2010, the number of consumers was limited for some of these products containing iodised salt (*n*<100), such as bakery mixtures, chips, sausage rolls and dressing. In combination with the low market shares containing iodised salt, these food groups were assumed to be all prepared without iodised salt. For the remaining food groups, a market share of 0·5 % was taken for meat products, Dutch rusk, other breakfast crackers, and 1 % was taken for cookies, cakes and sweet pies (Table 1).

The Dutch salt industry aims at an average I concentration of 20 mg/kg salt (low I salt) and for bakery salt an average of 58 mg/kg salt^(^ ^19^ ^)^. These concentrations were applied in our study.

### Dietary reference intakes for iodine

There are no national recommendations for I intake in the Netherlands. For this study, we used the US Institute of Medicine (currently Health and Medicine Division of the National Academies of Sciences, Engineering and Medicine) dietary reference values for I to evaluate the adequacy of I intake^(^ ^20^ ^)^. The EAR cutoff point method was used to estimate the proportion of the population with inadequate I intakes. The SCF (predecessor of the European Food and Safety Authority) has set a UL for I; these were used to estimate the proportion of the population with intakes above the UL^(^ ^2^ ^)^.

### Population characteristics

I intake can vary between population groups. The observed I intake from natural sources (excluding iodised salt and supplements) was estimated for several population characteristics (age, sex, BMI, income of the head of the household, education (for children, highest education of parents), season, region) using Proc SurveyMeans (SAS 9.2). With linear regression (Proc SurveyReg (SAS 9.2); *P*<0·05 statistically significant) it was studied how the intake of I naturally present in foods and population characteristics were associated, similar to Huybrechts *et al.* ^(^ ^26^ ^)^. These analyses were performed separately for children (7–18 years) and adults (19–69 years). The variables I intake and I intake/418 kJ (100 kcal) were natural log transformed to improve normality of the data and residuals. Potential outliers were detected and analyses were performed including and excluding these outliers to study the impact on the results. Linear regression was also performed to study the association between the intake of two food groups (milk and cheese) contributing most to the intake of I naturally present in foods (excluding iodised salt and supplements) and to the intake of bread (a main source of iodised salt), among consumers only. These intake variables were square root transformed to improve normality of the data and residuals, except for cheese intake among adults that was transformed with a natural log.

## Results

### Characteristics of the study population

The total study sample of 3819 subjects included 1713 children aged 7–18 years and 2106 adults (19–69 years) (Table 2). Compared with the Dutch population distribution, children were overrepresented and adults were underrepresented in the sample; however, with weighting for socio-demographic factors, this was corrected. In all, >40 % of the subjects originated from the western region, about 10 % from Northern Netherlands and 20–25 % from the East and the South; this represented the Netherlands well. The seasons were more or less equally distributed over the 1st recall days.

**Table 2:** Characteristics of the subjects participating in the Dutch National Food Consumption Survey 2007–2010 (Numbers and percentages)

	Children (7–18 years)				Adults (19–69 years)			
			Weighted†				Weighted†	
Characteristics*	*n*	%	*n*	%	*n*	%	*n*	%
*n*	1713		685		2106		3134	
Age–sex								
Boys (7–8 years)/men (19–30 years)	153	8·9	60	8·7	356	16·9	339	10·8
Girls (7–8 years)/women (19–30 years)	151	8·8	57	8·4	347	16·5	335	10·7
Boys (9–13 years)/men (31–50 years)	351	20·5	144	21·0	348	16·5	703	22·4
Girls (9–13 years)/women (31–50 years)	352	20·6	137	20·0	351	16·7	696	22·2
Boys (14–18 years)/men (51–69 years)	352	20·6	147	21·4	351	16·7	531	16·9
Girls (14–18 years)/women (51–69 years)	354	20·7	140	21·0	353	16·8	530	16·9
Region								
West	736	43·0	295	43·0	935	44·4	1401	44·7
North	180	10·5	72	10·5	218	10·4	325	10·4
East	379	22·1	155	22·7	446	21·2	657	21·0
South	418	24·4	164	23·9	507	24·1	751	24·0
Season (1st recall day)								
Spring	388	22·7	171	25·0	489	23·2	783	25·0
Summer	410	23·9	171	25·0	509	24·2	783	25·0
Autumn	433	25·3	171	25·0	517	24·6	784	25·0
Winter	482	28·1	171	25·0	591	28·1	783	25·0
Educational level								
Low	373	22·1	148	21·9	709	33·7	1007	32·1
Middle	725	42·9	294	43·5	935	44·4	136	7·6
High	591	35·0	233	34·6	462	21·9	761	24·3
Income head of house hold								
<Modal	634	37·0	256	37·3	779	37·0	1125	35·9
Modal–2× modal	974	56·9	390	56·9	1168	55·5	1747	55·8
>2× modal	105	6·1	40	5·8	159	7·6	261	8·3
BMI								
Underweight	156	9·1	63	9·3	48	2·3	56	1·8
Normal weight	1248	72·9	499	72·9	962	45·7	1370	43·8
Overweight	308	18·0	122	17·8	1095	52·0	1706	54·5

* BMI based on average height and weight (self-reported), categories based on^(^^9,^ ^11^ ^,^ ^12^ ^)^; education: low=primary school, lower vocational, low or intermediate general education; middle=intermediate vocational education and higher general education; high=higher vocational education and university; region west includes the three main cities of the Netherlands; modal income in 2010=32 500 € gross wage/year^(48)^.

† Weighted for socio-demographic factors to make samples representative of the Dutch population.

### Habitual iodine intake

The habitual I intake from natural sources only (excluding iodised salt and supplements) was, as expected, inadequate for most of the population (Table 3). For females aged 7–69 years, 84 % had an intake below their age-specific EAR; for males this was 67 %. For both males and females, the proportions were highest for adolescents and young adults (15–30 years).

**Table 3:** Estimated habitual total iodine intake from natural sources (excluding iodised salt and supplements) for Dutch men and women in several age categories and the proportion with a habitual intake below the estimated average requirement (EAR) (5th, 25th, 75th and 95th percentiles and medians)

		Habitual total I intake from natural sources (µg/d)						
	*n*	P5	P25	Median	P75	P95	EAR	%<EAR
All men (7–69 years)	1911	50	67	82	99	131	65–95	67
Boys (7–8 years)	153	36	47	56	68	89	65	70
Boys (9–13 years)	351	41	54	65	79	103	73	66
Boys (14–18 years)	352	47	61	74	89	116	95	82
Men (19–30 years)	356	52	68	82	98	129	95	71
Men (31–50 years)	348	55	72	87	105	137	95	62
Men (51–69 years)	351	53	70	84	101	132	95	67
All women (7–69 years)	1908	44	58	71	85	111	65–95	84
Girls (7–8 years)	151	36	47	56	67	87	65	71
Girls (9–13 years)	352	38	50	59	71	92	73	78
Girls (14–18 years)	354	40	53	63	75	97	95	94
Women (19–30 years)	347	43	56	67	80	104	95	90
Women (31–50 years)	351	47	61	73	87	112	95	84
Women (51–69 years)	353	49	64	77	92	118	95	79

In general, the habitual total I intake was higher for males compared with females and increased with age for both males and females, except men aged 51–69 years for whom the habitual intake was lower compared with men aged 31–50 years. (Table 4). For males, the median habitual total I intake ranged from 161 to 248 µg/d, for females the range of medians was 150–199 µg/d. The proportion with intakes below the EAR was 0–2 % in the different age groups and was slightly more frequent among females. Among females and adolescent and adult men the intake did not exceed the UL; for boys, 1–2 % had an intake above the UL (Table 4).

**Table 4:** Estimated habitual total iodine intake from foods, iodised salt and supplements (µg/d) for Dutch men and women in several age categories and the proportion with a habitual intake below the estimated average requirement (EAR) or above the tolerable upper intake level (UL) (Dutch National Food Consumption Survey 2007–2010) (Medians and standard deviations)

		Habitual total I intake (µg/d)															
			P5		P25				P75		P95			%<EAR			%>UL	
		*n*	Median	sd	Median	sd	Median	sd	Median	sd	Median	sd	EAR	Median	sd	UL	Median	sd
All men (7–69 years)		1911	138	0·9	194	1·0	233	1·3	274	1·6	341	2·2	65–95	1	0·0	300–600	1	0·1
Boys (7–8 years)		153	100	1·9	136	1·9	161	2·2	189	2·6	237	4·0	65	1	0·1	300	1	0·2
Boys (9–13 years)		351	120	1·4	164	1·3	194	1·5	227	1·8	282	2·8	73	0	0·1	300–450	2	0·2
Boys (14–18 years)		352	137	1·6	189	1·6	223	1·8	257	2·0	315	2·8	95	1	0·1	450–600	0	0·0
Men (19–30 years)		356	144	1·4	202	1·4	240	1·6	278	1·8	341	2·3	95	1	0·1	600	0	0·0
Men (31–50 years)		348	151	1·6	208	1·8	248	2·0	289	2·2	359	3·0	95	0	0·0	600	0	0·0
Men (51–69 years)		351	145	1·4	196	1·6	232	1·8	270	2·0	333	2·8	95	0	0·0	600	0	0·0
All women (7–69 years)		1908	117	0·6	158	0·6	188	0·8	219	1·1	277	1·7	65–95	1	0·0	300–600	0	0·1
Girls (7–8 years)		151	98	1·6	129	1·8	150	2·1	172	2·4	209	3·1	65	0	0·0	300	0	0·1
Girls (9–13 years)		352	106	1·1	141	1·1	164	1·2	189	1·5	229	2·1	73	1	0·1	300–450	0	0·0
Girls (14–18 years)		354	116	1·3	154	1·2	179	1·2	204	1·4	248	1·9	95	2	0·1	450–600	0	0·1
Women (19–30 years)		347	115	1·1	157	1·2	185	1·3	214	1·5	263	2·2	95	2	0·2	600	0	0·0
Women (31–50 years)		351	117	1·0	160	1·1	191	1·3	223	1·5	284	2·5	95	2	0·1	600	0	0·0
Women (51–69 years)		353	129	1·3	170	1·3	199	1·5	231	1·8	293	3·2	95	1	0·0	600	0	0·0

### Sources of iodine

**Fig. 2:**
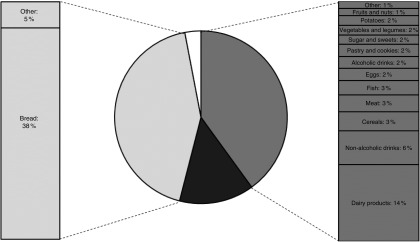
Average contribution of different sources to the total iodine intake for the Dutch population aged 7–69 years (Dutch National Food Consumption Survey 2007–2010). 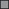, Naturally present 40 (sd 16) %; 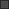, discretionarily iodised salt 14 (sd 12) %; 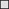, manufacturer iodised salt 43 (sd 19) %; 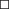, supplements 3 (sd 10) %.

On average, 40 % of the I intake came from I naturally present in food, 43 % came from iodised salt added by the food manufacturer, of which about 90 % could be contributed by bread prepared with iodised bakery salt. Discretionarily added iodised salt and supplements had an average contribution to the total I intake of 14 and 3 %, respectively (Fig. 2).

On average, the main sources of I naturally present in foods were dairy products (32 %), non-alcoholic drinks (14 %), cereals and cereal products (8 %), and meat and meat products (7 %) (Fig. 2). The contribution of the food groups was similar between children and adults, although children had a somewhat higher contribution from dairy products compared with adults (online Supplementary Table S1). Adult women had a slightly higher contribution from supplements. In the group of dairy products, milk (average 37–52 %) had the highest contribution to I intake for both children and adults followed by cheese (12–21 %) and yogurt (15–20 %), and also by milk-based drinks for children (12–16 %). Consumption of water contributed to about one-fourth of the I intake from non-alcoholic drinks. Diluted syrups and sodas had a high contribution for both children (50–58 %) and adults (23–31 %). For adults tea and coffee consumption also had a high contribution (37–40 %).

### Association with socio-demographic and lifestyle characteristics

Among children, the intake of I from natural sources was positively associated with sex (male) and region (west), and negatively associated with age (low *v*. high) and educational level of the parents (low *v*. high, online Supplementary Tables S2 and S3). After correction for energy intake, the associations with sex and age disappeared. This means that the differences in I intake from natural sources between sex and age categories could be explained by the differences in energy intake. Children of low-educated parents had a 10 % lower natural I intake compared with children of high-educated parents (63 *v*. 70 µg/d). This may partly be explained by an approximately 20 % higher intake of milk by children of high- *v*. low-educated parents (330 *v*. 396 g/d, online Supplementary Tables S6 and 7). Also, the proportion children not consuming milk was somewhat higher among children of middle-educated parents (30, 43, 27 %, respectively, low, middle and high; *P* 0·02). Children living in the western region of the Netherlands had a 6 % higher I intake compared with those living in the southern region (68 *v*. 64 µg/d); again, this may be partly explained by a higher consumption of milk: west, 391; and south, 352 g/d. The regional proportion of children not consuming milk was similar to the distribution of children living in specific regions in the Netherlands (data not shown). For adults, the intake of I from natural sources was associated with sex, age, educational level, region, season and income of the head of the household (online Supplementary Tables S2 and 3). After correction for energy intake, the associations with sex, age, region and educational level remained. In addition, an association with BMI appeared. In absolute quantities, the I intake was higher for males than females (88 *v*. 77 µg/d); however, relative to the energy intake, it was higher for females than males (4·0 *v*. 3·5 µg/418 kJ (100 kcal)). Similar to children, low-educated adults had a lower I intake compared with high-educated adults, which could partly be explained by an approximately 15 % lower consumption of cheese (cheese: 46 *v*. 53 g/d; online Supplementary Tables S6–S9) and a higher proportion of subjects not consuming cheese (33, 44, 23 %, respectively, low, middle and high educational level; *P*<0·0001). The average milk intake was similar between adults with different educational levels (335, 352, 345 ml/d for, respectively, low, middle and high educational level; online Supplementary Tables S6–S9). However, the proportion of low- and middle-educated adults was higher among those not consuming milk compared with high-educated adults (36, 45, 19 %, respectively; *P*<0·0001).

Among children, bread consumption, as proxy for the I intake from added iodised bakery salt, was associated with sex, age, BMI, income of the head of the household, education of the parents and season (online Supplementary Tables S4 and S5). After correction for energy intake, the associations with age, BMI, education of parents, and season remained. The older children (14–18 years, 144 g/d) consumed about 30 % more bread than younger children (7–8 years, 110 g/d); the largest part of this difference can be explained by an increased energy intake with age (data not shown). After correction for energy intake, bread consumption remained 8 % higher (data not shown). For adults, bread consumption was associated with sex, BMI, income of the head of the household and region (online Supplementary Tables S4 and S5). After correction for energy intake, the associations with the income of the head of the household and region maintained.

## Discussion

### Main results

The estimated habitual total I intake seems adequate and safe (i.e. between the EAR and UL) for the largest part of the general Dutch population (7–69 years). I naturally present in foods (excluding iodised salt and supplements) contributed to about 40 % of the total I intake, with dairy products and non-alcoholic drinks as the main sources. Another important source was iodised salt added by manufacturers (43 % of the estimated habitual total I intake), of which bread consumption contributed about 90 %. Children of low-educated parents had a 10 % lower I intake compared with children of high-educated parents, which is at least partly explained by a lower consumption of milk. In addition, these children consumed less bread, another important source of total I intake.

### Estimation of the iodine intake

Measuring I excretion in a repeated 24-h urine sample in combination with statistical correction for within-person variation is the golden standard for I status^(^ ^15^ ^–^ ^17^ ^)^. However, this only gives an insight into the actual I intake, without information on the dietary pattern. Detailed food consumption surveys provide an insight into the intake of nutrients and also into the important sources of this nutrient in the dietary pattern. In simulation studies, data from food consumption surveys can be used to study the effect of potential changes (e.g. I policy or food composition) on the intake, *a priori* ^(^ ^19^ ^)^. The total I intake is difficult to estimate from the Dutch food consumption surveys, similar to other consumption surveys, because of the several sources that are required to be taken into account and for some of which detailed data are lacking. Therefore, this study used a mathematical model, which is an improved version of an earlier model^(^ ^18^ ^,^ ^19^ ^)^. In contrast to the earlier model, the habitual total I intake distribution was estimated with a ‘first shrink then add’ approach instead of a ‘first add then shrink’ approach^(^ ^24^ ^,^ ^27^ ^–^ ^31^ ^)^. An advantage of this approach is that potential differences in model parameters for the several sources of total I intake – like within- and between-person variations, transformation – are retained in the modelling. In addition, it is possible to take into account source-specific information on non-users (e.g. relevant for supplements and discretionary use of salt). All of these may influence the distribution and the evaluation of whether the intake is adequate or safe.

Further, detailed data on the use of iodised salt by manufacturers as well as discretionary use are lacking. Additional information was collected to overcome this issue; however, several assumptions had to be made as well. Food manufacturers (members of FNLI and NVB) provided data on the use of iodised salt in their products, and foods containing iodised salt as an ingredient were searched for in a commercial food database (www.innovadatabase.com). Altogether, this gives a more or less complete image of the use of iodised salt by manufacturers of foods available on the Dutch market. This inventory showed that the use of iodised salt by manufacturers is limited, except for bread and, to a lesser extent, pizza. On the basis of this information, assumptions regarding the market share of products within food groups containing iodised salt were decreased compared with earlier assumptions. For the I policy change in 2008 it was assumed that 50 % of the foods, other than bread, would be prepared with iodised salt^(^ ^14^ ^)^; our study showed that in 2012 this was a large overestimation. Consequently, the proposed population I intake could not be obtained. In addition, the 5 % assumed in later scenario studies seemed already an overestimation^(^ ^18^ ^,^ ^19^ ^)^. Iodised salt is added to a limited part of the food groups, with a limited market share.

Information on the discretionary use of salt was collected in the 3rd year of data collection of DNFCS 2007–2010; the age and sex-specific proportion of subjects using discretionarily iodised salt was applied to the data of the 1st and 2nd year of data collection. This only provided information on whether subjects were using discretionarily iodised salt; it was not known in what foods and in what amount. On the basis of other studies, assumptions were made. For better estimation of the I intake, it is important to collect detailed information on the discretionary use of iodised salt: for what foods or dishes it is used, what amounts are added, and in line with that, how much is retained in the food consumed.

Similar to the earlier model^(^ ^18^ ^,^ ^19^ ^)^, the results of our modelling exercise were compared with the I intake estimated from single 24-h urine samples in the Doetinchem Cohort Study, a town in the eastern part of the Netherlands^(^ ^13^ ^)^ (data not shown). The I intakes estimated in our study were somewhat higher, but in the same order of magnitude, compared with the I intake estimated from urine samples. This indicates that the calculation model is good to estimate the I intake. The differences may be due to the several assumptions in the mathematical model, but also to differences between the populations in both studies, for example, national representative *v*. one city, age distribution.

### Iodine concentration in food and supplements

Similar to food composition tables in other countries, the Dutch food composition database contains nutrient information from chemical analyses, but also information from, for example, foreign databases, recipe calculations and label information. For our study, the I concentration data were updated, compared with earlier studies^(^ ^18^ ^,^ ^19^ ^)^; however, continuous updates are required to maintain good data quality. The I content of food depends on the I content in soil and feed; therefore, large differences may appear within food groups produced in different places in the world. The impact of this on the Dutch food supply is unclear. Recent studies from UK show that organic milk has lower I concentrations than conventional milk, and that winter milk contains higher I concentration compared with summer milk^(^ ^32^ ^,^ ^33^ ^)^. For bread, there may also be a difference in I content between organic and conventional; because of a court decision, the addition of iodised bakery salt is not mandatory in the Netherlands. However, via a covenant between the Dutch Ministry of Health and manufacturers of conventional bread, the use of iodised bakery salt is encouraged, resulting in virtually all conventional fresh bread being baked with iodised bakery salt. Organic and imported breads (e.g. partially baked bread segment) are not included in this covenant. As a consequence, not all organic bread and imported bread contain iodised bakery salt. This may implicate that subjects consuming organic foods may be at risk of inadequate I intakes. The organic food market is growing in the Netherlands^(^ ^34^ ^)^. As such, the number of subjects consuming bread without iodised bakery salt may increase. Unfortunately, from the DNFCS 2007–2010, it is not clear who consumed organic foods. It is important to get a better insight into the consumption pattern of those consuming organic foods, as well as into the range of I concentrations within food groups, especially between organic and conventional foods. Another important issue is the worldwide effort to reduce salt intake; as fortification with I is mainly via iodisation of salt, reduction of salt intake may influence the I intake^(^ ^35^ ^–^ ^38^ ^)^. To maintain adequate I intake, it may be required to increase salt I levels or search for other ways to elevate the I intake and/or content of foods in a safe way.

The information on the label was used for the composition information of dietary supplements. Italian research showed that part of the I-containing supplements contained higher levels than declared^(^ ^39^ ^)^. It is unknown how well the label information regarding I content of supplements in the Netherlands predicts the actual concentration. The use of I-containing supplements is rather low in our population (about 10 %) and the potential effect of underestimation or overestimation is expected to be limited. To improve the nutrient estimations, it is recommended to study the actual nutrient content, including I, of supplements.

### Comparison with other research

I intake in the Netherlands depends largely on the I policy. This policy changed in 2008. It is therefore impossible to check the validity of our calculation model with studies on I intake in the Netherlands before 2008. Further, international differences in I policy also influence the interpretation of comparisons of I intake between countries. The results of the 24-h urine sample study were used to validate the mathematical models; it is therefore expected that our study also shows a reduced I intake compared with earlier estimations^(^ ^18^ ^)^. The effects of a changing I policy^(^ ^10^ ^)^ mainly cause this reduction. In this policy, the I concentration for bread was reduced in order to maintain safe I intakes when a much larger part of the food supply (50 %) would contain iodised salt. As explained earlier, this assumption appeared to be an overestimation of the situation in 2012.

Similar to our study, dairy products were an important I source in Iceland^(^ ^40^ ^)^ and Norway^(^ ^41^ ^)^, and non-alcoholic beverages in Germany^(^ ^42^ ^)^. Because of the Dutch I policy, most of the bread contains iodised bakery salt, which has a higher I level than do other foods and household salt. This makes bread an important source of I in the Netherlands. Also, in some other countries, bread with iodised salt is used as vehicle to increase I intake^(^ ^43^ ^–^ ^46^ ^)^. In the Netherlands, for a long time, I intake was considered adequate, but transferring the Dutch I policy to other countries will not always result in adequate I intakes, because of differences in food habits. This is also important to consider in the ongoing EU harmonisation for addition of vitamins and minerals to foods^(^ ^47^ ^)^ – especially for I, a nutrient for which many countries rely on addition to food to get a population with adequate intakes.

### Implications for research

There are several methodological challenges in the estimation of the habitual I intake and its sources. For I, similar to other nutrients like Na, more information is required than currently present in food consumption surveys. Some suggestions for improvement of data collection will be presented. First, if there is a mixture of foods within a product category being or not being produced with iodised salt, it is important to know which food was consumed exactly and whether this was produced with iodised salt. In addition, the composition of these foods should be available. Second, information on discretionary use of iodised salt is required. The 24-h recall (or other methods) could be expanded with specific questions on whether iodised salt was used during meal preparation or consumption so that information can be gathered on specific food groups. Third, an additional requirement is knowledge on the amounts of iodised salt discretionarily added and the retention during meal preparation. It is very difficult, perhaps impossible, to ask subjects to quantify how much salt they added. Additional research chemically measuring the amount of I in food after a normal meal preparation would probably give a more reliable result. However, people are aware of the advice to restrict salt, which may influence the results if subjects know the topic of research. Further, to get an insight into the variation of addition of salt, it is important to conduct this research in a large group, preferably representative of the population. Fourth, to validate a calculation model, information from a golden standard is required. It is best to have both available from the same subjects in the same time period.

### Conclusions

The I intake of the general population in the Netherlands, aged 7–69 years, seems adequate, although lower compared with that during the period before 2008. Our study did not include all age groups, and no pregnant or lactating women; conclusions on adequacy may not be valid for those groups. The same is true for subjects with thyroid-related diseases. Iodised bakery salt added to bread, as well as dairy products and non-alcoholic drinks, are important sources of I. With the current effort to reduce the salt intake and changing dietary patterns (i.e. less bread, more organic foods) it is important to keep a close track on the I status, important sources and potential risk groups, and, when needed, to adapt the I policy in order to maintain adequacy of the I intake in the Netherlands.

## Supplementary material

For supplementary material accompanying this paper visit http://dx.doi.org/10.1017/S0007114517000733. click here to view supplementary material
