# Supplementary material for: Decreased, but still sufficient, iodine intake of children and adults in the Netherlands
Source: Br J Nutr. 2017 Apr 14;117(7):1020–31. doi: 10.1017/S0007114517000733 (PMC5448465; doi:10.1017/S0007114517000733)
Supplement: Supplementary file 1 [file S0007114517000733sup001.docx]

**Supplemental Tables S1-S9: Iodine intake of children and adults in the Netherlands**

**Table S.1** Average contribution of food groups to the intake of total iodine by age and gender (DNFCS 2007-2010)

|  | | |  | Average contribution (%) | | | | | | | | | | |
| --- | --- | --- | --- | --- | --- | --- | --- | --- | --- | --- | --- | --- | --- | --- |
|  | | |  | 7-8 yrs. | 9-13 yrs. | | 14-18 yrs. | | 19-30 yrs. | | 31-50 yrs. | | 51-69 yrs. | |
|  | | |  |  | men | women | men | women | men | women | men | women | men | women |
| Naturally present in foods | | | | 40 | 38 | 41 | 37 | 37 | 38 | 41 | 38 | 42 | 40 | 42 |
|  | Dairy | | | 37 | 35 | 33 | 29 | 32 | 34 | 36 | 33 | 31 | 32 | 34 |
|  | Non-alcoholic drinks | | | 12 | 13 | 15 | 15 | 13 | 10 | 13 | 16 | 17 | 16 | 13 |
|  | Cereals | | | 8 | 8 | 9 | 9 | 8 | 7 | 8 | 9 | 8 | 7 | 6 |
|  | Meat | | | 7 | 7 | 8 | 8 | 8 | 8 | 7 | 8 | 7 | 6 | 6 |
|  | Fish | | | 4 | 5 | 3 | 5 | 7 | 7 | 4 | 3 | 5 | 6 | 8 |
|  | Eggs | | | 4 | 5 | 4 | 6 | 4 | 5 | 4 | 4 | 6 | 4 | 6 |
|  | Alcoholic drinks | | | 0 | 0 | 2 | 5 | 6 | 8 | 0 | 1 | 2 | 4 | 8 |
|  | Pastry & cookies | | | 6 | 6 | 5 | 4 | 4 | 4 | 6 | 6 | 5 | 5 | 4 |
|  | Sugar & sweets | | | 8 | 8 | 6 | 4 | 3 | 3 | 8 | 6 | 5 | 4 | 3 |
|  | Vegetables & legumes | | | 3 | 3 | 3 | 3 | 4 | 4 | 3 | 4 | 4 | 4 | 4 |
|  | Potatoes | | | 4 | 4 | 5 | 4 | 4 | 4 | 4 | 4 | 3 | 3 | 3 |
|  | Fruits & nuts | | | 4 | 3 | 2 | 2 | 3 | 3 | 3 | 3 | 3 | 3 | 4 |
|  | | | |  |  |  |  |  |  |  |  |  |  |  |
| Added by manufacturer | | | | 48 | 47 | 46 | 46 | 46 | 46 | 41 | 45 | 41 | 42 | 37 |
|  | Bread | | | 91 | 90 | 90 | 89 | 90 | 87 | 85 | 89 | 86 | 90 | 88 |
|  | |  | |  |  |  |  |  |  |  |  |  |  |  |
| Discretionally added | |  | | 11 | 13 | 11 | 15 | 14 | 14 | 14 | 13 | 12 | 16 | 15 |
|  | |  | |  |  |  |  |  |  |  |  |  |  |  |
| Dietary supplements | |  | | 1 | 1 | 1 | 1 | 2 | 2 | 3 | 3 | 5 | 2 | 5 |

**Table S.2** Average observed (mean over two recall days) iodine intake naturally present in foods (excluding iodized salt and dietary supplements) by population characteristics and season, weighted for socio-demographic factors (DNFCS-2007-2010; N=3.819, 7-69 yrs.)

| **Characteristics^*^** | |  | **Iodine intake** | | | | | | |
| --- | --- | --- | --- | --- | --- | --- | --- | --- | --- |
|  |  |  | **Children (7-18 yrs.)** | | |  | **Adults (19-69 yrs.)** | | |
|  |  |  | **n** | **µg/d (SE)** | **µg/100 kcal/d (SE)** |  | **n** | **µg/d (SE)** | **µg/100 kcal/d (SE)** |
| Total |  |  | 1713 | 66 (0.7) | 3.1 (0.0) |  | 2106 | 83 (0.9) | 3.8 (0.0) |
| Gender | Men |  | 856 | 70 (1.0) | 3.0 (0.0) |  | 1055 | 88 (1.4) | 3.5 (0.1) |
|  | Women |  | 857 | 61 (0.9) | 3.1 (0.1) |  | 1051 | 77 (1.1) | 4.0 (0.1) |
| BMI^†^ | Underweight |  | 156 | 61 (2.4) | 2.9 (0.1) |  | 48 | 83 (5.2) | 3.4 (0.2) |
|  | Normal weight |  | 1248 | 67 (0.8) | 3.1 (0.0) |  | 962 | 82 (1.3) | 3.7 (0.1) |
|  | Overweight |  | 308 | 64 (1.6) | 3.2 (0.1) |  | 1095 | 83 (1.3) | 3.9 (0.1) |
| Income head of household | < modal |  | 634 | 64 (1.2) | 3.0 (0.1) |  | 779 | 82 (1.5) | 3.8 (0.1) |
|  | Modal-2x modal |  | 974 | 67 (0.9) | 3.1 (0.0) |  | 1168 | 82 (1.1) | 3.7 (0.0) |
|  | > 2x modal |  | 105 | 66 (2.3) | 3.1 (0.1) |  | 159 | 89 (3.4) | 4.1 (0.2) |
| Education^‡^ | Low |  | 373 | 63 (1.6) | 2.9 (0.1) |  | 709 | 80 (1.5) | 3.7 (0.1) |
|  | Middle |  | 725 | 65 (1.0) | 3.0 (0.0) |  | 935 | 83 (1.3) | 3.7 (0.1) |
|  | High |  | 591 | 70 (1.3) | 3.3 (0.1) |  | 462 | 87 (2.1) | 4.0 (0.1) |
| Age | 7-8 yrs. / 19-30 yrs. |  | 304 | 58 (1.6) | 3.1 (0.1) |  | 703 | 80 (1.4) | 3.5 (0.1) |
|  | 9-13 yrs. / 31-50 yrs. |  | 703 | 65 (1.1) | 3.0 (0.0) |  | 699 | 82 (1.5) | 3.7 (0.1) |
|  | 14-18 yrs. / 51-69 yrs. |  | 706 | 70 (1.1) | 3.1 (0.1) |  | 704 | 85 (1.5) | 4.1 (0.1) |
| Region | West |  | 736 | 68 (1.1) | 3.1 (0.0) |  | 935 | 85 (1.4) | 3.8 (0.1) |
|  | North |  | 180 | 63 (1.9) | 2.9 (0.1) |  | 218 | 85 (2.7) | 3.7 (0.1) |
|  | East |  | 379 | 66 (1.5) | 3.1 (0.1) |  | 446 | 82 (1.9) | 3.8 (0.1) |
|  | South |  | 418 | 64 (1.3) | 2.9 (0.1) |  | 507 | 78 (1.8) | 3.7 (0.1) |
| Season | Spring |  | 388 | 67 (1.5) | 3.1 (0.1) |  | 489 | 83 (1.8) | 3.8 (0.1) |
|  | Summer |  | 410 | 66 (1.4) | 3.1 (0.1) |  | 509 | 84 (1.7) | 3.8 (0.1) |
|  | Autumn |  | 433 | 64 (1.3) | 3.0 (0.1) |  | 517 | 82 (1.8) | 3.8 (0.1) |
|  | Winter |  | 482 | 67 (1.4) | 3.0 (0.1) |  | 591 | 81 (1.7) | 3.7 (0.1) |

^*^ BMI based on average height and weight (self-reported), categories based on ^(47; 48; 49)^; education low = primary school, lower vocational, low or intermediate general education, middle = intermediate vocational education and higher general education, high= higher vocational education and university; region west includes the three main cities of the Netherlands; modal income in 2010 = 32.500 euro gross wage per year ^(50)^

^†^ 1 missing value children, 1 missing value adults

^‡^ 24 missing values children

**Table S.3.** Association between iodine intake from natural sources (excl. iodized salt and dietary supplements) and socio-demographic characteristics, weighted to socio-demographic factors (DNFCS-2007-2010; N=3.819, 7-69 yrs.), statistical significant associations (P<0.05) in bold.

| **Characteristic*^,†^** | | **Children (7-18 yrs.) (n=1688)^‡^** | | | | | |  | **Adults (19-69 yrs.) (n=2105) ^‡^** | | | | |
| --- | --- | --- | --- | --- | --- | --- | --- | --- | --- | --- | --- | --- | --- |
|  |  | **Iodine intake** | | | | | |  | **Iodine intake** | | | | |
|  |  | **µg/d** | | |  | **µg/100kcal/d** | |  | **µg/d** | |  | **µg/100kcal/d** | |
|  |  | **β (SE)** | | **p-value** |  | **β (SE)** | **p-value** |  | **β (SE)** | **p-value** |  | **β (SE)** | **p-value** |
| Intercept | **4.08 (0.05)** | | **<0.0001** | |  | **1.09 (0.05)** | **<0.0001** |  | **4.30 (0.05)** | **<0.0001** |  | **1.49 (0.04)** | **<0.0001** |
| Gender (men) | **0.13 (0.02)** | | **<0.0001** | |  | -0.03 (0.02) | 0.058 |  | **0.13 (0.02)** | **<0.0001** |  | **-0.16 (0.02)** | **<0.0001** |
| Age group (7-8 yrs./19-30 yrs.) | **-0.19 (0.03)** | | **<0.0001** | |  | -0.004 (0.03) | 0.879 |  | **-0.08 (0.02)** | **0.0008** |  | **-0.18 (0.02)** | **<0.0001** |
| Age group (9-13 yrs./31-50 yrs.) | **-0.06 (0.02)** | | **0.004** | |  | 0.001 (0.02) | 0.947 |  | **-0.06 (0.02)** | **0.013** |  | **-0.12 (0.02)** | **<0.0001** |
| BMI – underweight | -0.06 (0.04) | | 0.149 | |  | -0.06 (0.04) | 0.085 |  | 0.06 (0.07) | 0.360 |  | -0.08 (0.06) | 0.131 |
| BMI – normal weight | 0.02 (0.03) | | 0.412 | |  | -0.01 (0.03) | 0.566 |  | -0.0002 (0.02) | 0.991 |  | **-0.05 (0.02)** | **0.013** |
| Income head house hold (low) | 0.02 (0.04) | | 0.553 | |  | 0.01 (0.04) | 0.706 |  | **-0.08 (0.04)** | **0.047** |  | -0.06 (0.04) | 0.121 |
| Income head house hold (middle) (midden) | 0.05 (0.04) | | 0.226 | |  | 0.02 (0.03) | 0.542 |  | -0.06 (0.04) | 0.072 |  | -0.06 (0.03) | 0.103 |
| Education (low) | **-0.10 (0.03)** | | **0.0004** | |  | **-0.09 (0.03)** | **0.0005** |  | **-0.07 (0.03)** | **0.017** |  | **-0.11 (0.02)** | **<0.0001** |
| Education (middle) | **-0.05 (0.02)** | | **0.021** | |  | **-0.06 (0.02)** | **0.005** |  | -0.01 (0.03) | 0.566 |  | **-0.05 (0.02)** | **0.019** |
| Region (west) | **0.05 (0.02)** | | **0.035** | |  | **0.05 (0.02)** | **0.027** |  | **0.09 (0.02)** | **0.0004** |  | **0.06 (0.02)** | **0.004** |
| Region (east) | 0.003 (0.04) | | 0.938 | |  | -0.004 (0.03) | 0.888 |  | **0.10 (0.03)** | **0.004** |  | 0.04 (0.03) | 0.237 |
| Region (north) | 0.03 (0.03) | | 0.275 | |  | 0.02 (0.03) | 0.349 |  | **0.06 (0.03)** | **0.030** |  | 0.05 (0.03) | 0.073 |
| Season (spring) | -0.004 (0.03) | | 0.875 | |  | 0.005 (0.02) | 0.840 |  | 0.04 (0.03) | 0.187 |  | 0.03(0.02) | 0.210 |
| Season (summer) | -0.02 (0.03) | | 0.357 | |  | -0.0004 (0.03) | 0.988 |  | **0.06 (0.03)** | **0.040** |  | 0.04 (0.02) | 0.142 |
| Season (autumn) | -0.04 (0.03) | | 0.068 | |  | -0.02 (0.02) | 0.409 |  | 0.02 (0.03) | 0.572 |  | 0.03 (0.033) | 0.316 |

^*^ BMI based on average height and weight (self-reported), categories based on ^(47; 48; 49)^; education low = primary school, lower vocational, low or intermediate general education, middle = intermediate vocational education and higher general education, high= higher vocational education and university; region west includes the three main cities of the Netherlands; modal income in 2010 = 32.500 euro gross wage per year ^(50)^

^†^ Reference groups are: gender: female; age group child: 14-18 yrs., adult: 51-69 yrs.; BMI: overweight; income head of household: high; educational level: high; region: south; season: winter

^‡^ 25 children and 1 adult had missing values in at least one characteristic

**Table S.4** Average observed (mean over two recall days) bread consumption by population characteristics and season, weighted for socio-demographic factors (DNFCS-2007-2010; N=3.819, 7-69 yrs.)

| **Characteristics*** | |  | **Bread consumption** | | | | |
| --- | --- | --- | --- | --- | --- | --- | --- |
|  |  |  | **Children (7-18 yrs.)** | |  | **Adults (19-69 yrs.)** | |
|  |  |  | **n** | **g/d (SE)** |  | **n** | **g/d (SE)** |
| Total |  |  | 1700 | 129 (1.6) |  | 2070 | 137 (1.5) |
| Gender | Men |  | 851 | 143(2.5) |  | 1036 | 159 (2.4) |
|  | Women |  | 849 | 115 (1.8) |  | 1034 | 114 (1.7) |
| BMI^†^ | Underweight |  | 154 | 124(5.0) |  | 48 | 148 (12.7) |
|  | Normal weight |  | 1240 | 129 (1.9) |  | 944 | 141 (2.4) |
|  | Overweight |  | 305 | 132 (3.8) |  | 1077 | 133 (2.0) |
| Income head of household | < modal |  | 630 | 125 (2.6) |  | 761 | 135 (2.5) |
|  | Modal-2x modal |  | 965 | 132 (2.2) |  | 1155 | 140 (2.1) |
|  | > 2x modal |  | 105 | 123 (5.2) |  | 154 | 126 (5.6) |
| Education^‡^ | Low |  | 369 | 124 (3.5) |  | 698 | 133 (2.6) |
|  | Middle |  | 720 | 126 (2.4) |  | 917 | 141 (2.3) |
|  | High |  | 587 | 137 (2.8) |  | 455 | 133 (3.3) |
| Age | 7-8 yrs. / 19-30 yrs. |  | 303 | 110 (3.1) |  | 690 | 144 (2.9) |
|  | 9-13 yrs. / 31-50 yrs. |  | 697 | 122 (2.3) |  | 688 | 138 (2.4) |
|  | 14-18 yrs. / 51-69 yrs. |  | 700 | 144 (2.7) |  | 692 | 131 (2.4) |
| Region | West |  | 730 | 128 (2.5) |  | 916 | 137 (2.3) |
|  | North |  | 178 | 126 (4.3) |  | 217 | 127 (4.5) |
|  | East |  | 375 | 129 (3.6) |  | 437 | 134 (3.5) |
|  | South |  | 417 | 132 (3.1) |  | 500 | 143 (3.1) |
| Season | Spring |  | 382 | 131(3.2) |  | 483 | 140 (3.1) |
|  | Summer |  | 407 | 124 (3.2) |  | 500 | 137 (2.9) |
|  | Autumn |  | 430 | 136 (3.4) |  | 508 | 133 (3.1) |
|  | Winter |  | 481 | 125 (3.1) |  | 579 | 137 (3.3) |

^*^ BMI based on average height and weight (self-reported), categories based on ^(47; 48; 49)^; education low = primary school, lower vocational, low or intermediate general education, middle = intermediate vocational education and higher general education, high= higher vocational education and university; region west includes the three main cities of the Netherlands; modal income in 2010 = 32.500 euro gross wage per year ^(50)^

^†^ 1 missing value children, 1 missing value adults

^‡^ 24 missing values children

**Table S.5** Association between bread consumption and socio-demographic characteristics, weighted to socio-demographic factors (DNFCS-2007-2010; N=3.819, 7-69 yrs.), statistical significant associations (P<0.05) in bold.

| **Characteristic^*,†^** | **Children (7-18 yrs.) (n=1675)^‡^** | | | | |  | **Adults (19-69 yrs.) (n=2069)^‡^** | | | | |
| --- | --- | --- | --- | --- | --- | --- | --- | --- | --- | --- | --- |
|  | **Bread consumption** | | | | |  | **Bread consumption** | | | | |
|  | **g/d** | |  | **g/100kcal/d** | |  | **g/d** | |  | **g/100kcal/d** | |
|  | **β (SE)** | **p-value** |  | **β (SE)** | **p-value** |  | **β (SE)** | **p-value** |  | **β (SE)** | **p-value** |
| Intercept | **11.17 (0.35) (7.8)** | **<0.0001** |  | **2.49 (0.07)** | **<0.0001** |  | **9.58 (0.30)** | **<0.0001** |  | **2.36 (0.06)** | **<0.0001** |
| Gender (men) | **1.16 (0.14)** | **<0.0001** |  | 0.04 (0.03) | 0.105 |  | **1.93 (0.13)** | **<0.0001** |  | 0.05 (0.03) | 0.062 |
| Age group (7-8 yrs./19-30 yrs.) | **-1.41 (0.18)** | **<0.0001** |  | **-0.08 (0.04)** | **0.041** |  | 0.29 (0.17) | 0.076 |  | -0.06 (0.03) | 0.075 |
| Age group (9-13 yrs./31-50 yrs.) | **-0.85 (0.15)** | **<0.0001** |  | **-0.09 (0.03)** | **0.002** |  | 0.26 (0.15) | 0.077 |  | -0.02 (0.03) | 0.430 |
| BMI – underweight | -0.51 (0.26) | 0.052 |  | **-0.12 (0.05)** | **0.027** |  | 0.76 (0.53) | 0.150 |  | 0.02 (0.12) | 0.869 |
| BMI – normal weight | **-0.43 (0.18)** | **0.015** |  | **-0.13 (0.04)** | **0.0003** |  | **0.36 (0.13)** | **0.007** |  | 0.01 (0.03) | 0.724 |
| Income head house hold (low) | 0.54 (0.29) | 0.062 |  | 0.08 (0.06) | 0.159 |  | 0.47 (0.26) | 0.073 |  | **0.11 (0.06)** | **0.042** |
| Income head house hold (middle) (midden) | **0.58 (0.27)** | **0.031** |  | 0.08 (0.06) | 0.158 |  | **0.59 (0.25)** | **0.017** |  | **0.13 (0.05)** | **0.014** |
| Education (low) | **-0.68 (0.19)** | **0.0005** |  | **-0.13 (0.04)** | **0.001** |  | 0.17 (0.18) | 0.359 |  | -0.01 (0.04) | 0.857 |
| Education (middle) | **-0.53 (0.16)** | **0.001** |  | **-0.12 (0.03)** | **0.0002** |  | 0.32 (0.17) | 0.066 |  | 0.03 (0.04) | 0.472 |
| Region (west) | -0.15 (0.17) | 0.369 |  | -0.04 (0.03) | 0.287 |  | **-0.28 (0.15)** | **0.064** |  | **-0.09 (0.03)** | **0.006** |
| Region (east) | -0.11 (0.23) | 0.610 |  | -0.04 (0.05) | 0.448 |  | **-0.68 (0.23)** | **0.003** |  | **-0.20 (0.05)** | **<0.0001** |
| Region (north) | -0.09 (0.20) | 0.660 |  | -0.03 (0.04) | 0.388 |  | **-0.45 (0.19)** | **0.016** |  | **-0.11 (0.04)** | **0.004** |
| Season (spring) | 0.28 (0.19) | 0.140 |  | **0.08 (0.04)** | **0.039** |  | 0.21 (0.18) | 0.245 |  | 0.05 (0.04) | 0.164 |
| Season (summer) | -0.09 (0.20) | 0.656 |  | 0.01 (0.04) | 0.874 |  | 0.06 (0.18) | 0.758 |  | -0.003 (0.04) | 0.926 |
| Season (autumn) | **0.47 (0.19)** | **0.014** |  | **0.12 (0.04)** | **0.001** |  | -0.09 (0.19) | 0.614 |  | -0.01 (0.04) | 0.759 |

^*^ BMI based on average height and weight (self-reported), categories based on ^(47; 48; 49)^; education low = primary school, lower vocational, low or intermediate general education, middle = intermediate vocational education and higher general education, high= higher vocational education and university; region west includes the three main cities of the Netherlands; modal income in 2010 = 32.500 euro gross wage per year ^(50)^

^†^ Reference groups are: gender: female; age group child: 14-18 yrs., adult: 51-69 yrs.; BMI: overweight; income head of household: high; educational level: high; region: south; season: winter

^‡^ 25 children and 1 adult had missing values in at least one characteristic

**Table S.6** Average observed (mean over two recall days) milk consumption by population characteristics and season, weighted for socio-demographic factors (DNFCS-2007-2010; N=3.819, 7-69 yrs.)

| **Characteristics*** | |  | **Milk consumption** | | | | |
| --- | --- | --- | --- | --- | --- | --- | --- |
|  |  |  | **Children (7-18 yrs.)** | |  | **Adults (19-69 yrs.)** | |
|  |  |  | **n** | **ml/d (SE)** |  | **n** | **ml/d (SE)** |
| Total |  |  | 1523 | 371 (7) |  | 1753 | 345 (7) |
| Gender | Men |  | 765 | 388 (9) |  | 883 | 378 (11) |
|  | Women |  | 758 | 353 (9) |  | 870 | 311 (8) |
| BMI^†^ | Underweight |  | 135 | 333 (20) |  | 37 | 319 (38) |
|  | Normal weight |  | 1122 | 378 (8) |  | 809 | 340 (10) |
|  | Overweight |  | 265 | 361 (17) |  | 906 | 350 (9) |
| Income head of household | < modal |  | 555 | 349 (10) |  | 645 | 338 (12) |
|  | Modal-2x modal |  | 873 | 384 (9) |  | 972 | 352 (9) |
|  | > 2x modal |  | 95 | 378 (23) |  | 136 | 325 (25) |
| Education^‡^ | Low |  | 318 | 330 (12) |  | 583 | 335 (11) |
|  | Middle |  | 646 | 371 (10) |  | 776 | 352 (10) |
|  | High |  | 540 | 396 (11) |  | 394 | 345 (15) |
| Age | 7-8 yrs. / 19-30 yrs. |  | 274 | 381 (15) |  | 576 | 350 (12) |
|  | 9-13 yrs. / 31-50 yrs. |  | 642 | 364 (10) |  | 591 | 343 (11) |
|  | 14-18 yrs. / 51-69 yrs. |  | 607 | 373 (11) |  | 586 | 344 (11) |
| Region | West |  | 658 | 391 (10) |  | 783 | 353 (10) |
|  | North |  | 166 | 359 (20) |  | 186 | 376 (22) |
|  | East |  | 337 | 356 (12) |  | 383 | 354 (14) |
|  | South |  | 362 | 352 (13) |  | 401 | 304 (14) |
| Season | Spring |  | 344 | 360 (14) |  | 409 | 350 (16) |
|  | Summer |  | 360 | 348 (12) |  | 422 | 343 (13) |
|  | Autumn |  | 387 | 377 (12) |  | 433 | 339 (12) |
|  | Winter |  | 432 | 398 (14) |  | 489 | 348 (14) |

^*^ BMI based on average height and weight (self-reported), categories based on ^(47; 48; 49)^; education low = primary school, lower vocational, low or intermediate general education, middle = intermediate vocational education and higher general education, high= higher vocational education and university; region west includes the three main cities of the Netherlands; modal income in 2010 = 32.500 euro gross wage per year ^(50)^

^†^ 1 missing value children, 1 missing value adults

^‡^ 24 missing values children

**Table S.7** Association between milk consumption and socio-demographic characteristics, weighted to socio-demographic factors (DNFCS-2007-2010; N=3.819, 7-69 yrs.), statistical significant associations (P<0.05) in bold.

| **Characteristic*^,†^** | **Children (7-18 yrs.) (n=1503)^‡^** | | | | | |  | **Adults (19-69 yrs.) (n=1752)^‡^** | | | | |
| --- | --- | --- | --- | --- | --- | --- | --- | --- | --- | --- | --- | --- |
|  | **Milk consumption** | | | | | |  | **Milk consumption** | | | | |
|  | **ml/d** | | |  | **ml/100kcal/d** | |  | **ml/d** | |  | **ml/100kcal/d** | |
|  | **β (SE)** | | **p-value** |  | **β (SE)** | **p-value** |  | **β (SE)** | **p-value** |  | **β (SE)** | **p-value** |
| Intercept | | **18.03 (0.87)** | **<0.0001** |  | **3.96 (0.19)** | **<0.0001** |  | **14.68 (30)** | **<0.0001** |  | **3.59 (0.18)** | **<0.0001** |
| Gender (men) | | **0.90 (0.34)** | **0.008** |  | -0.5 (0.6) | 0.357 |  | **1.42 (0.36)** | **<0.0001** |  | **-0.24 (0.07)** | **0.001** |
| Age group (7-8 yrs./19-30 yrs.) | | 0.49 (0.50) | 0.323 |  | **0.50 (0.11)** | **<0.0001** |  | -0.01 (0.46) | 0.988 |  | -0.18 (0.10) | 0.059 |
| Age group (9-13 yrs./31-50 yrs.) | | 0.21 (0.38) | 0.573 |  | **0.20 (0.08)** | **0.014** |  | -0.03 (0.42) | 0.939 |  | -0.12 (0.09) | 0.178 |
| BMI – underweight | | -0.84 (0.71) | 0.238 |  | -0.19 (0.15) | 0.225 |  | -0.20 (1.18) | 0.865 |  | -0.30 (0.22) | 0.184 |
| BMI – normal weight | | 0.45 (0.49) | 0.352 |  | 0.06 (0.10) | 0.582 |  | -0.15 (0.37) | 0.691 |  | -0.12 (0.08) | 0.130 |
| Income head house hold (low) | | -0.44 (0.71) | 0.531 |  | -0.12 (0.15) | 0.415 |  | 0.37 (0.73) | 0.612 |  | 0.08 (0.15) | 0.580 |
| Income head house hold (middle) (midden) | | 0.21 (0.66) | 0.745 |  | -0.01 (0.14) | 0.970 |  | 0.86 (0.68) | 0.210 |  | 0.17 (0.14) | 0.213 |
| Education (low) | | **-1.47 (0.48)** | **0.002** |  | **-0.30 (0.10)** | **0.004** |  | -0.05 (0.51) | 0.915 |  | -0.07 (0.11) | 0.509 |
| Education (middle) | | -0.53 (0.41) | 0.192 |  | -0.14 (0.09) | 0.107 |  | 0.23 (0.49) | 0.642 |  | 0.01 (0.10) | 0.910 |
| Region (west) | | **1.00 (0.43)** | **0.021** |  | **0.21 (0.09)** | **0.023** |  | **1.54 (0.46)** | **0.0009** |  | **0.28 (0.10)** | **0.004** |
| Region (east) | | 0.47 (0.63) | 0.457 |  | 0.08 (0.13) | 0.523 |  | **2.29 (0.66)** | **0.0006** |  | **0.41 (0.13)** | **0.003** |
| Region (north) | | 0.27 (0.49) | 0.591 |  | 0.03 (0.11) | 0.777 |  | **1.63 (0.53)** | **0.0021** |  | **0.33 (0.11)** | **0.003** |
| Season (spring) | | **-1.12 (0.50)** | **0.026** |  | **-0.21 (0.10)** | **0.041** |  | 0.04 (0.53) | 0.934 |  | 0.01 (0.11) | 0.931 |
| Season (summer) | | **-1.23 (0.47)** | **0.009** |  | **-0.20 (0.10)** | **0.048** |  | -0.05 (0.49) | 0.915 |  | -0.01 (0.10) | 0.920 |
| Season (autumn) | | -0.49 (0.47) | 0.296 |  | -0.06 (0.10) | 0.551 |  | -0.11 (0.48) | 0.820 |  | -0.01 (0.10) | 0.925 |

^*^ BMI based on average height and weight (self-reported), categories based on ^(47; 48; 49)^; education low = primary school, lower vocational, low or intermediate general education, middle = intermediate vocational education and higher general education, high= higher vocational education and university; region west includes the three main cities of the Netherlands; modal income in 2010 = 32.500 euro gross wage per year ^(50)^

^†^ Reference groups are: gender: female; age group child: 14-18 yrs., adult: 51-69 yrs.; BMI: overweight; income head of household: high; educational level: high; region: south; season: winter

^‡^ 25 children and 1 adult had missing values in at least one characteristic

**Table S.8** Average observed (mean over two recall days) cheese consumption by population characteristics and season, weighted for socio-demographic factors (DNFCS-2007-2010; N=3.819, 7-69 yrs.)

| **Characteristics^*^** | |  | **Cheese consumption** | | | | |  |
| --- | --- | --- | --- | --- | --- | --- | --- | --- |
|  |  |  | **Children (7-18 yrs.)** | |  | **Adults (19-69 yrs.)** | | |
|  |  |  | **n** | **g/d (SE)** |  | **n** | **g/d (SE)** | |
| Total |  |  | 1244 | 38 (1.0) |  | 1766 | 50 (1.1) | |
| Gender | Men |  | 604 | 40 (1.5) |  | 863 | 53 (1.6) | |
|  | Women |  | 640 | 35 (1.2) |  | 903 | 47 (1.5) | |
| BMI^†^ | Underweight |  | 101 | 39 (3.4) |  | 41 | 66 (9) | |
|  | Normal weight |  | 913 | 37 (1.2) |  | 800 | 49 (1.7) | |
|  | Overweight |  | 229 | 39 (1.9) |  | 924 | 51 (1.5) | |
| Income head of household | < modal |  | 456 | 35 (1.5) |  | 648 | 51 (1.9) | |
|  | Modal-2x modal |  | 707 | 39 (1.3) |  | 986 | 49 (1.4) | |
|  | > 2x modal |  | 81 | 41 (4.4) |  | 132 | 53 (4.7) | |
| Education^‡^ | Low |  | 255 | 38 (2.3) |  | 597 | 46 (1.7) | |
|  | Middle |  | 522 | 36 (1.3) |  | 784 | 52 (1.7) | |
|  | High |  | 447 | 39 (1.8) |  | 385 | 53 (2.4) | |
| Age | 7-8 yrs. / 19-30 yrs. |  | 218 | 33 (1.9) |  | 566 | 50 (1.9) | |
|  | 9-13 yrs. / 31-50 yrs. |  | 510 | 35 (1.4) |  | 571 | 50 (1.8) | |
|  | 14-18 yrs. / 51-69 yrs. |  | 516 | 42 (1.7) |  | 629 | 51 (1.9) | |
| Region | West |  | 538 | 38 (1.5) |  | 793 | 52 (1.8) | |
|  | North |  | 122 | 34 (2.5) |  | 181 | 49 (3.8) | |
|  | East |  | 275 | 39 (2.4) |  | 364 | 47 (2.2) | |
|  | South |  | 309 | 36 (1.7) |  | 428 | 49 (2.0) | |
| Season | Spring |  | 275 | 38 (2.2) |  | 410 | 49 (2.2) | |
|  | Summer |  | 308 | 38 (2.2) |  | 420 | 51 (2.2) | |
|  | Autumn |  | 314 | 37 (1.8) |  | 439 | 50 (2.2) | |
|  | Winter |  | 347 | 37 (1.6) |  | 497 | 49 (2.2) | |

^*^ BMI based on average height and weight (self-reported), categories based on ^(47; 48; 49)^; education low = primary school, lower vocational, low or intermediate general education, middle = intermediate vocational education and higher general education, high= higher vocational education and university; region west includes the three main cities of the Netherlands; modal income in 2010 = 32.500 euro gross wage per year ^(50)^

^†^ 1 missing value children, 1 missing value adults

^‡^ 24 missing values children

**Table S.9** Association between cheese consumption and socio-demographic characteristics, weighted to socio-demographic factors (DNFCS-2007-2010; N=3.819, 7-69 yrs.), statistical significant associations (P<0.05) in bold.

| **Characteristic^*,†^** | **Children (7-18 yrs.) (n=1244)^‡^** | | | | |  | **Adults (19-69 yrs.) (n=1765) ^‡^** | | | | |
| --- | --- | --- | --- | --- | --- | --- | --- | --- | --- | --- | --- |
|  | **Cheese consumption** | | | | |  | **Cheese consumption** | | | | |
|  | **g/d** | |  | **g/100kcal/d** | |  | **g/d** | |  | **g/100kcal/d** | |
|  | **β (SE)** | **p-value** |  | **β (SE)** | **p-value** |  | **β (SE)** | **p-value** |  | **β (SE)** | **p-value** |
| Intercept | **5.98 (0.36)** | **<0.0001** |  | **1.33 (0.08)** | **<0.0001** |  | **3.67 (0.10)** | **<0.0001** |  | **0.85 (0.11)** | **<0.0001** |
| Gender (men) | **0.48 (0.14)** | **0.0006** |  | -0.003 (0.03) | 0.926 |  | **0.15 (0.04)** | **0.0004** |  | **-0.14 (0.04)** | **0.001** |
| Age group (7-8 yrs./19-30 yrs.) | **-0.68 (0.20)** | **0.0006** |  | -0.04 (0.04) | 0.360 |  | -0.08 (0.05) | 0.116 |  | **-0.17 (0.05)** | **0.001** |
| Age group (9-13 yrs./31-50 yrs.) | **-0.48 (0.16)** | **0.002** |  | -0.06 (0.03) | 0.078 |  | -0.06 (0.05) | 0.214 |  | **-0.12 (0.05)** | **0.012** |
| BMI – underweight | -0.21 (0.28) | 0.446 |  | -0.05 (0.07) | 0.485 |  | **0.31 (0.14)** | **0.026** |  | 0.14 (0.14) | 0.339 |
| BMI – normal weight | **-0.35 (0.17)** | **0.037** |  | **-0.10 (0.04)** | **0.005** |  | -0.06 (0.04) | 0.145 |  | **-0.11 (0.04)** | **0.010** |
| Income head house hold (low) | -0.20 (0.33) | 0.545 |  | -0.07 (0.07) | 0.337 |  | 0.01 (0.08) | 0.937 |  | 0.003 (0.8) | 0.967 |
| Income head house hold (middle) (midden) | -0.01 (0.32) | 0.964 |  | -0.04 (0.07) | 0.531 |  | -0.03 (0.08) | 0.699 |  | -0.03 (0.08) | 0.709 |
| Education (low) | 0.10 (0.21) | 0.623 |  | -0.03 (0.041) | 0.492 |  | **-0.15 (0.06)** | **0.010** |  | **-0.19 (0.06)** | **0.001** |
| Education (middle) | -0.08 (0.17) | 0.620 |  | -0.01 (0.04) | 0.743 |  | -0.04 (0.06) | 0.471 |  | -0.09 (0.06) | 0.114 |
| Region (west) | 0.11 (0.17) | 0.531 |  | 0.041 (0.04) | 0.281 |  | -0.001 (0.05) | 0.991 |  | -0.03 (0.05) | 0.584 |
| Region (east) | -0.10 (0.23) | 0.675 |  | -0.003 (0.05) | 0.948 |  | -0.10 (0.08) | 0.252 |  | -0.15 (0.09) | 0.087 |
| Region (north) | 0.13 (0.21) | 0.523 |  | 0.03 (0.04) | 0.436 |  | -0.10 (0.06) | 0.136 |  | -0.11 (0.06) | 0.066 |
| Season (spring) | 0.11 (0.20) | 0.578 |  | 0.03 (0.04) | 0.403 |  | 0.04 (0.06) | 0.462 |  | 0.05 (0.06) | 0.393 |
| Season (summer) | 0.05 (0.19) | 0.794 |  | 0.02 (0.04) | 0.589 |  | 0.08 (0.06) | 0.161 |  | 0.07 (0.06) | 0.239 |
| Season (autumn) | 0.11 (0.19) | 0.568 |  | 0.04 (0.04) | 0.352 |  | 0.05 (0.06) | 0.393 |  | 0.05 (0.06) | 0.380 |

^*^ BMI based on average height and weight (self-reported), categories based on ^(47; 48; 49)^; education low = primary school, lower vocational, low or intermediate general education, middle = intermediate vocational education and higher general education, high= higher vocational education and university; region west includes the three main cities of the Netherlands; modal income in 2010 = 32.500 euro gross wage per year ^(50)^

^†^ Reference groups are: gender: female; age group child: 14-18 yrs., adult: 51-69 yrs.; BMI: overweight; income head of household: high; educational level: high; region: south; season: winter

^‡^ 25 children and 1 adult had missing values in at least one characteristic
